# Bis[μ-2-(4-hy­droxy­phen­yl)acetato]-κ^3^ *O*,*O*′:*O*;κ^3^ *O*:*O*,*O*′-bis­{aqua­(4,4′-bi­pyridine-κ*N*)[2-(4-hy­droxy­phen­yl)acetato-κ^2^ *O*,*O*′]erbium(III)} monohydrate

**DOI:** 10.1107/S1600536810044752

**Published:** 2010-11-06

**Authors:** Jia-Lu Liu, Jian-Feng Liu, Guo-Liang Zhao

**Affiliations:** a College of Chemistry and Life Sciences, Zhejiang Normal University, Jinhua 321004, People’s Republic of China, and Zhejiang Normal University Xingzhi College, Jinhua 321004, People’s Republic of China

## Abstract

The title dinuclear complex, [Er_2_(C_8_H_7_O_3_)_6_(C_10_H_8_N_2_)_2_(H_2_O)_2_]·H_2_O, contains two Er^III^ atoms in the asymmetric unit, six 2-(4-hy­droxy­phen­yl)acetate (PAA) anions, two 4,4′-bipyridine (bipy) mol­ecules, two coordinated water mol­ecules and one solvent water mol­ecule. The central Er^III^ atoms are nine-coordinated. The coordination environment includes six O atoms from three PAA anions, one bridging O atom from a PAA anion, one O atom from a water mol­ecule and an N atom from a bipy ligand in a distorted tricapped trigonal–prismatic geometry. The occurrence of numerous O—H⋯O and O—H⋯N hydrogen bonds involving coordinated and noncoordinated water as well as bipy groups builds up an intricate three-dimensional network.

## Related literature

For the crystal structure of a related thulium compound with 1,10-phenanthroline instead of 4,4′-bipyridine, see: Liu *et al.* (2010 ▶).
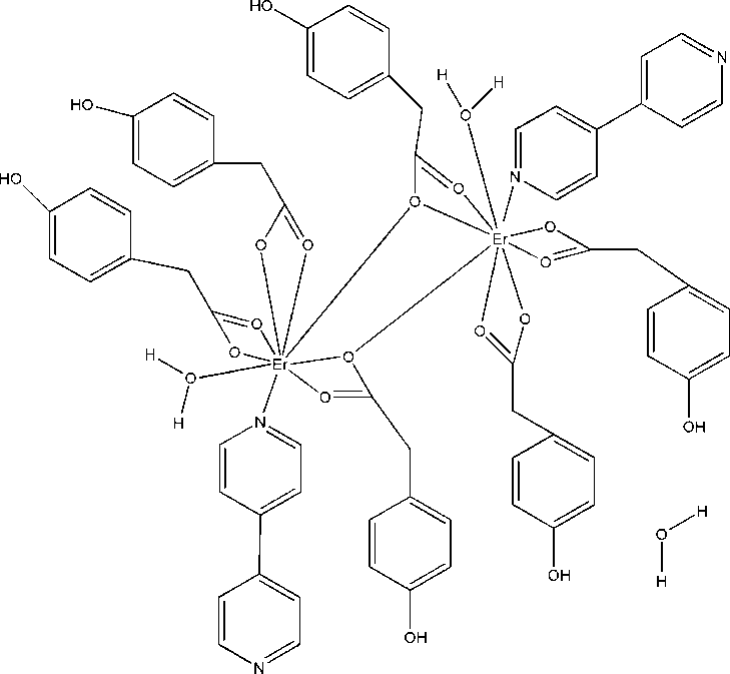


## Experimental

### 

#### Crystal data

[Er_2_(C_8_H_7_O_3_)_6_(C_10_H_8_N_2_)_2_(H_2_O)_2_]·H_2_O
*M* *_r_* = 1607.76
Triclinic, 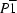

*a* = 11.7043 (2) Å
*b* = 16.1714 (3) Å
*c* = 18.4117 (3) Å
α = 83.350 (1)°
β = 72.339 (1)°
γ = 71.359 (1)°
*V* = 3145.74 (10) Å^3^
*Z* = 2
Mo *K*α radiation
μ = 2.73 mm^−1^
*T* = 296 K
0.19 × 0.15 × 0.10 mm

#### Data collection

Bruker APEXII diffractometer
Absorption correction: multi-scan (*SADABS*; Sheldrick, 1996 ▶) *T*_min_ = 0.620, *T*_max_ = 0.761
47710 measured reflections
14291 independent reflections
12810 reflections with *I* > 2σ(*I*)
*R*_int_ = 0.020

#### Refinement

*R*[*F* ^2^ > 2σ(*F* ^2^)] = 0.018
*wR*(*F* ^2^) = 0.044
*S* = 1.03
14291 reflections
875 parameters
9 restraints
H atoms treated by a mixture of independent and constrained refinement
Δρ_max_ = 0.41 e Å^−3^
Δρ_min_ = −0.80 e Å^−3^

### 

Data collection: *APEX2* (Bruker, 2006 ▶); cell refinement: *SAINT* (Bruker, 2006 ▶); data reduction: *SAINT*; program(s) used to solve structure: *SHELXS97* (Sheldrick, 2008 ▶); program(s) used to refine structure: *SHELXL97* (Sheldrick, 2008 ▶); molecular graphics: *SHELXTL* (Sheldrick, 2008 ▶); software used to prepare material for publication: *SHELXL97*.

## Supplementary Material

Crystal structure: contains datablocks I, global. DOI: 10.1107/S1600536810044752/om2372sup1.cif

Structure factors: contains datablocks I. DOI: 10.1107/S1600536810044752/om2372Isup2.hkl

Additional supplementary materials:  crystallographic information; 3D view; checkCIF report

## Figures and Tables

**Table 1:** Hydrogen-bond geometry (Å, °)

*D*—H⋯*A*	*D*—H	H⋯*A*	*D*⋯*A*	*D*—H⋯*A*
O3—H3*B*⋯O12^i^	0.82	1.93	2.742 (3)	169
O6—H6*B*⋯O3*W*^ii^	0.82	1.86	2.650 (3)	160
O9—H9*A*⋯O17^iii^	0.82	1.86	2.674 (2)	173
O12—H12⋯O11^iv^	0.82	1.94	2.751 (2)	168
O15—H15*C*⋯O6^v^	0.82	1.90	2.716 (3)	174
O18—H18*B*⋯O9^ii^	0.82	1.95	2.768 (3)	174
O2*W*—H2*WA*⋯O5	0.83 (4)	1.97 (2)	2.732 (2)	151 (3)
O2*W*—H2*WB*⋯N2^ii^	0.81 (2)	2.06 (2)	2.839 (2)	162 (4)
O3*W*—H3*WB*⋯O3	0.82 (4)	1.99 (4)	2.793 (3)	167 (4)
O1*W*—H1*WA*⋯O13	0.83 (4)	1.96 (2)	2.7333 (19)	156 (3)
O1*W*—H1*WB*⋯N4^i^	0.83 (2)	1.96 (2)	2.781 (2)	171 (3)
O3*W*—H3*WA*⋯O1^vi^	0.84 (4)	1.94 (2)	2.778 (3)	176 (4)

Symmetry codes: (i) 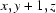; (ii) 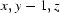; (iii) 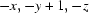; (iv) 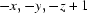; (v) 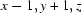; (vi) 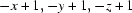.

**Fig. 1.**
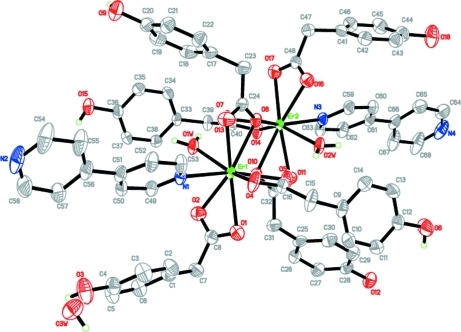
The molecular structure of the title complex, showing the atom-labelling scheme. Displacement ellipsoids are drawn at the 50% probability level (The H atoms not involved in hydrogen bonding were omitted for clarity).

## supplementary crystallographic information

### Comment

The design and synthesis of lanthanide carboxylic metal–organic complexes has been of interest for decades owing to their potential practical applications, such as fluorescence and magnetism. We recently published the structure of a thulium(III) complex with coordination provided by 2-(4-hydroxyphenyl)acetate and 1,10-phenanthroline. (Liu *et al.*, 2010). In this paper, we report the related crystal structure of a new erbium(III) complex with the ligand 2-(4-hydroxyphenyl)acetate and 4,4'-bipyridine instead of 1,10-phenathroline. The dinuclear complex, [Er(C_8_H_7_O_3_)_3_(C_10_H_8_N_2_) (H_2_0)]_2_.(H_2_0), contains two erbium(III) ions in the asymmetric unit, six 2-(4-hydroxyphenyl)acetate (PAA) anions, two 4,4'-bipyridine molecules (bipy), as well as two coordinated water molecules. There is one solvate water molecule. The central Er ions are nine coordinate. The have similar coordination spheres, which include six O atoms from three 2-(4-hydroxyphenyl)acetate ligands, one bridging O atom from a neighboring PAA ligand, one O atom from a water molecule and a N atom from bipy ligand in a distorted tricapped trigonal-prismatic geometry. In the species, the Er's are coordinated by two modes, bridging and bridging tridentate (Fig. 1). Each Er is in a distorted tricapped trigonal-prismatic geometry. Futhermore, the asymmetric unit contains one solvent water molecules (Fig. 1). The occurrence of numerous O—H···O and O—H···N hydrogen bonds involving coordinated and non-coordinated water as well as bipyridine groups builds up an intricate three-dimensionnal network (Table 1).

### Experimental

All reagents and solvents used were of commercially available quality and were used without further purification. Para-hydroxyphenylacetic acid (HPAA) (3 mmol) and sodium hydroxide (3 mmol) were mixed together in water (10 ml), then Er[(NO_3_)_3_] (1 mmol) dissolved in water (10 ml) was added into the above solution. After stirring for one hour, an ethanol (5 ml) solution of 4,4'-bipyridine (1 mmol) was slowly dripped into the above solution with stirring for three hours. After filtration, the filtrate was allowed to stand at room temperature, and single crystals suitable for X-ray work were obtained after one week.

### Refinement

All H atoms attached to C atoms and O(hydroxyl) atom were fixed geometrically and treated as riding with C—H = 0.97 Å (methylene) or 0.93 Å (aromatic) and O—H = 0.82 Å with *U*_iso_(H) = 1.2*U*_eq_(C) or *U*_iso_(H) = 1.5*U*_eq_(O). H atoms of the water molecule were located in a difference Fourier map and included in the subsequent refinement using restraints (O—H = 0.82 (1) Å and H···H= 1.39 (2) Å) with *U*_iso_(H) = 1.5*U*_eq_(O). In the last cycles of refinement they were treated as riding on their parent O atom.

### Crystal data

**Table:**

[Er_2_(C_8_H_7_O_3_)_6_(C_10_H_8_N_2_)_2_(H_2_O)_2_]·H_2_O	*Z* = 2
*M**_r_* = 1607.76	*F*(000) = 1608
Triclinic, *P*1	*D*_x_ = 1.697 Mg m^−^^3^
Hall symbol: -P 1	Mo *K*α radiation, λ = 0.71073 Å
*a* = 11.7043 (2) Å	Cell parameters from 9914 reflections
*b* = 16.1714 (3) Å	θ = 1.8–27.7°
*c* = 18.4117 (3) Å	µ = 2.73 mm^−^^1^
α = 83.350 (1)°	*T* = 296 K
β = 72.339 (1)°	Block, brown
γ = 71.359 (1)°	0.19 × 0.15 × 0.10 mm
*V* = 3145.74 (10) Å^3^	

### Data collection

**Table:**

Bruker APEXII diffractometer	14291 independent reflections
Radiation source: fine-focus sealed tube	12810 reflections with *I* > 2σ(*I*)
graphite	*R*_int_ = 0.020
φ and ω scans	θ_max_ = 27.7°, θ_min_ = 1.8°
Absorption correction: multi-scan (*SADABS*; Sheldrick, 1996)	*h* = −15→15
*T*_min_ = 0.620, *T*_max_ = 0.761	*k* = −21→21
47710 measured reflections	*l* = −24→24

### Refinement

**Table:**

Refinement on *F*^2^	Primary atom site location: structure-invariant direct methods
Least-squares matrix: full	Secondary atom site location: difference Fourier map
*R*[*F*^2^ > 2σ(*F*^2^)] = 0.018	Hydrogen site location: inferred from neighbouring sites
*wR*(*F*^2^) = 0.044	H atoms treated by a mixture of independent and constrained refinement
*S* = 1.03	*w* = 1/[σ^2^(*F*_o_^2^) + (0.0177*P*)^2^ + 1.8781*P*] where *P* = (*F*_o_^2^ + 2*F*_c_^2^)/3
14291 reflections	(Δ/σ)_max_ = 0.002
875 parameters	Δρ_max_ = 0.41 e Å^−^^3^
9 restraints	Δρ_min_ = −0.79 e Å^−^^3^

### Special details

**Table:**

Geometry. All e.s.d.'s (except the e.s.d. in the dihedral angle between two l.s. planes) are estimated using the full covariance matrix. The cell e.s.d.'s are taken into account individually in the estimation of e.s.d.'s in distances, angles and torsion angles; correlations between e.s.d.'s in cell parameters are only used when they are defined by crystal symmetry. An approximate (isotropic) treatment of cell e.s.d.'s is used for estimating e.s.d.'s involving l.s. planes.
Refinement. Refinement of *F*^2^ against ALL reflections. The weighted *R*-factor *wR* and goodness of fit *S* are based on *F*^2^, conventional *R*-factors *R* are based on *F*, with *F* set to zero for negative *F*^2^. The threshold expression of *F*^2^ > σ(*F*^2^) is used only for calculating *R*-factors(gt) *etc*. and is not relevant to the choice of reflections for refinement. *R*-factors based on *F*^2^ are statistically about twice as large as those based on *F*, and *R*- factors based on ALL data will be even larger.

### Fractional atomic coordinates and isotropic or equivalent isotropic displacement parameters (Å^2^)

**Table:**

	*x*	*y*	*z*	*U*_iso_*/*U*_eq_	
Er1	0.272626 (7)	0.363745 (5)	0.282022 (5)	0.02268 (3)	
Er2	0.131164 (7)	0.207912 (5)	0.197904 (5)	0.02233 (3)	
N1	0.31200 (15)	0.50792 (10)	0.28188 (10)	0.0307 (4)	
N2	0.3925 (2)	0.92876 (14)	0.22401 (18)	0.0643 (7)	
N3	0.08811 (16)	0.06535 (10)	0.19105 (10)	0.0298 (4)	
N4	0.0361 (2)	−0.36355 (13)	0.22761 (17)	0.0626 (7)	
C1	0.2256 (2)	0.50765 (14)	0.51543 (12)	0.0334 (4)	
C2	0.3418 (2)	0.51383 (15)	0.47165 (14)	0.0412 (5)	
H2	0.3984	0.4669	0.4422	0.049*	
C3	0.3753 (2)	0.58859 (16)	0.47094 (15)	0.0453 (6)	
H3	0.4536	0.5918	0.4408	0.054*	
C4	0.2932 (2)	0.65817 (15)	0.51468 (14)	0.0400 (5)	
C5	0.1783 (2)	0.65263 (15)	0.56002 (15)	0.0446 (6)	
H5	0.1234	0.6989	0.5909	0.054*	
C6	0.1447 (2)	0.57835 (15)	0.55962 (14)	0.0413 (5)	
H6	0.0661	0.5756	0.5896	0.050*	
C7	0.1869 (2)	0.42605 (15)	0.51792 (13)	0.0390 (5)	
H7A	0.2319	0.3805	0.5471	0.047*	
H7B	0.0977	0.4386	0.5434	0.047*	
C8	0.2142 (2)	0.39421 (13)	0.43890 (12)	0.0331 (4)	
C9	0.62243 (19)	0.09048 (13)	0.30165 (14)	0.0361 (5)	
C10	0.6034 (3)	0.05923 (15)	0.37554 (15)	0.0485 (6)	
H10	0.5904	0.0966	0.4140	0.058*	
C11	0.6029 (3)	−0.02615 (15)	0.39469 (14)	0.0475 (6)	
H11	0.5895	−0.0456	0.4452	0.057*	
C12	0.6224 (2)	−0.08167 (13)	0.33793 (13)	0.0360 (5)	
C13	0.6402 (2)	−0.05169 (15)	0.26370 (14)	0.0437 (5)	
H13	0.6525	−0.0890	0.2253	0.052*	
C14	0.6399 (2)	0.03333 (15)	0.24595 (14)	0.0432 (5)	
H14	0.6518	0.0529	0.1955	0.052*	
C15	0.6248 (2)	0.18284 (14)	0.28110 (18)	0.0479 (6)	
H15A	0.6902	0.1825	0.2337	0.058*	
H15B	0.6476	0.2040	0.3203	0.058*	
C16	0.50311 (18)	0.24622 (12)	0.27212 (12)	0.0295 (4)	
C17	0.2730 (2)	0.45397 (14)	0.01310 (12)	0.0394 (5)	
C18	0.1471 (2)	0.50128 (17)	0.02819 (13)	0.0451 (6)	
H18	0.0866	0.4745	0.0549	0.054*	
C19	0.1091 (2)	0.58785 (16)	0.00428 (14)	0.0429 (5)	
H19	0.0240	0.6187	0.0150	0.051*	
C20	0.1987 (2)	0.62797 (14)	−0.03568 (13)	0.0364 (5)	
C21	0.3247 (2)	0.58185 (14)	−0.05022 (14)	0.0390 (5)	
H21	0.3852	0.6088	−0.0763	0.047*	
C22	0.3609 (2)	0.49584 (14)	−0.02606 (14)	0.0396 (5)	
H22	0.4461	0.4654	−0.0363	0.048*	
C23	0.3133 (3)	0.36015 (15)	0.04024 (14)	0.0541 (7)	
H23A	0.2593	0.3298	0.0317	0.065*	
H23B	0.3986	0.3319	0.0105	0.065*	
C24	0.3073 (2)	0.35285 (12)	0.12354 (12)	0.0304 (4)	
C25	0.1423 (2)	0.11464 (14)	0.47612 (12)	0.0350 (5)	
C26	0.0399 (2)	0.10725 (15)	0.53574 (13)	0.0402 (5)	
H26	−0.0287	0.1565	0.5514	0.048*	
C27	0.0380 (2)	0.02738 (15)	0.57261 (13)	0.0397 (5)	
H27	−0.0311	0.0234	0.6128	0.048*	
C28	0.1386 (2)	−0.04556 (13)	0.54937 (12)	0.0332 (4)	
C29	0.2423 (2)	−0.03927 (15)	0.49027 (13)	0.0394 (5)	
H29	0.3108	−0.0885	0.4747	0.047*	
C30	0.2433 (2)	0.04070 (15)	0.45467 (13)	0.0400 (5)	
H30	0.3135	0.0448	0.4154	0.048*	
C31	0.1444 (3)	0.19981 (14)	0.43375 (13)	0.0415 (5)	
H31A	0.0805	0.2468	0.4647	0.050*	
H31B	0.2253	0.2083	0.4274	0.050*	
C32	0.12247 (18)	0.20650 (12)	0.35667 (12)	0.0281 (4)	
C33	−0.25296 (17)	0.47932 (12)	0.24488 (12)	0.0282 (4)	
C34	−0.2103 (2)	0.51785 (14)	0.17454 (13)	0.0352 (5)	
H34	−0.1721	0.4834	0.1315	0.042*	
C35	−0.2233 (2)	0.60557 (14)	0.16719 (13)	0.0378 (5)	
H35	−0.1947	0.6300	0.1195	0.045*	
C36	−0.2787 (2)	0.65764 (13)	0.23040 (13)	0.0350 (5)	
C37	−0.3217 (2)	0.62089 (13)	0.30101 (13)	0.0379 (5)	
H37	−0.3594	0.6555	0.3439	0.045*	
C38	−0.3084 (2)	0.53220 (13)	0.30768 (13)	0.0346 (5)	
H38	−0.3374	0.5079	0.3554	0.042*	
C39	−0.24006 (18)	0.38311 (12)	0.25171 (14)	0.0346 (5)	
H39A	−0.2849	0.3703	0.3032	0.042*	
H39B	−0.2806	0.3702	0.2175	0.042*	
C40	−0.10703 (18)	0.32306 (12)	0.23417 (11)	0.0272 (4)	
C41	0.2330 (2)	0.05678 (14)	−0.04405 (12)	0.0374 (5)	
C42	0.3521 (2)	0.00595 (15)	−0.04052 (13)	0.0391 (5)	
H42	0.4083	0.0334	−0.0359	0.047*	
C43	0.3895 (2)	−0.08380 (15)	−0.04370 (14)	0.0423 (5)	
H43	0.4702	−0.1162	−0.0420	0.051*	
C44	0.3063 (2)	−0.12537 (15)	−0.04945 (14)	0.0447 (5)	
C45	0.1879 (2)	−0.07640 (16)	−0.05424 (15)	0.0468 (6)	
H45	0.1320	−0.1040	−0.0591	0.056*	
C46	0.1527 (2)	0.01357 (16)	−0.05181 (14)	0.0432 (5)	
H46	0.0731	0.0459	−0.0555	0.052*	
C47	0.1901 (3)	0.15508 (15)	−0.03735 (13)	0.0435 (5)	
H47A	0.2530	0.1788	−0.0725	0.052*	
H47B	0.1124	0.1793	−0.0515	0.052*	
C48	0.1693 (2)	0.18189 (12)	0.04274 (12)	0.0336 (5)	
C49	0.2288 (2)	0.57667 (13)	0.32171 (13)	0.0339 (4)	
H49	0.1554	0.5693	0.3557	0.041*	
C50	0.2461 (2)	0.65824 (13)	0.31501 (13)	0.0353 (5)	
H50	0.1857	0.7041	0.3442	0.042*	
C51	0.3543 (2)	0.67110 (13)	0.26442 (13)	0.0337 (5)	
C52	0.4421 (2)	0.59934 (14)	0.22450 (15)	0.0403 (5)	
H52	0.5170	0.6046	0.1909	0.048*	
C53	0.4180 (2)	0.52015 (13)	0.23478 (14)	0.0380 (5)	
H53	0.4785	0.4728	0.2077	0.046*	
C54	0.4495 (3)	0.86765 (18)	0.1723 (2)	0.0736 (10)	
H54	0.4982	0.8824	0.1255	0.088*	
C55	0.4421 (2)	0.78325 (16)	0.18269 (19)	0.0596 (8)	
H55	0.4835	0.7434	0.1436	0.072*	
C56	0.3724 (2)	0.75902 (13)	0.25202 (15)	0.0397 (5)	
C57	0.3164 (3)	0.82154 (16)	0.30725 (18)	0.0601 (8)	
H57	0.2712	0.8080	0.3557	0.072*	
C58	0.3277 (3)	0.90479 (18)	0.2903 (2)	0.0691 (9)	
H58	0.2869	0.9463	0.3281	0.083*	
C59	0.1750 (2)	−0.00035 (13)	0.14942 (13)	0.0361 (5)	
H59	0.2488	0.0093	0.1182	0.043*	
C60	0.1609 (2)	−0.08242 (13)	0.15041 (14)	0.0401 (5)	
H60	0.2235	−0.1257	0.1193	0.048*	
C61	0.0545 (2)	−0.10022 (12)	0.19732 (13)	0.0343 (5)	
C62	−0.0381 (2)	−0.03086 (14)	0.23823 (14)	0.0396 (5)	
H62	−0.1135	−0.0386	0.2690	0.048*	
C63	−0.0185 (2)	0.04953 (13)	0.23336 (14)	0.0361 (5)	
H63	−0.0825	0.0951	0.2609	0.043*	
C64	0.1158 (4)	−0.33945 (17)	0.16761 (19)	0.0669 (9)	
H64	0.1706	−0.3818	0.1327	0.080*	
C65	0.1225 (3)	−0.25481 (15)	0.15369 (17)	0.0588 (8)	
H65	0.1793	−0.2413	0.1100	0.071*	
C66	0.0440 (2)	−0.19045 (13)	0.20547 (15)	0.0418 (6)	
C67	−0.0408 (2)	−0.21508 (16)	0.2679 (2)	0.0581 (8)	
H67	−0.0965	−0.1744	0.3041	0.070*	
C68	−0.0415 (3)	−0.30106 (17)	0.2758 (2)	0.0690 (10)	
H68	−0.1002	−0.3161	0.3175	0.083*	
O1W	0.08967 (14)	0.45713 (9)	0.25376 (10)	0.0351 (3)	
O1	0.31321 (16)	0.33485 (10)	0.41030 (10)	0.0448 (4)	
O2W	0.32191 (14)	0.11389 (9)	0.21641 (10)	0.0365 (3)	
O2	0.13911 (14)	0.42916 (10)	0.39984 (9)	0.0384 (3)	
O3	0.32934 (18)	0.73159 (12)	0.51239 (13)	0.0621 (5)	
H3B	0.2710	0.7695	0.5383	0.053 (8)*	
O3W	0.55572 (19)	0.76710 (13)	0.49150 (12)	0.0557 (5)	
O4	0.49306 (14)	0.32552 (9)	0.26308 (10)	0.0423 (4)	
O5	0.41097 (13)	0.21949 (9)	0.27590 (9)	0.0355 (3)	
O6	0.6256 (2)	−0.16741 (10)	0.35221 (10)	0.0575 (5)	
H6B	0.5986	−0.1755	0.3982	0.086*	
O7	0.35154 (16)	0.39907 (10)	0.14982 (9)	0.0419 (4)	
O8	0.25588 (12)	0.30030 (8)	0.16729 (8)	0.0288 (3)	
O9	0.16710 (17)	0.71316 (10)	−0.06077 (11)	0.0515 (4)	
H9A	0.0962	0.7273	−0.0660	0.077*	
O10	0.14490 (12)	0.26954 (8)	0.31222 (8)	0.0276 (3)	
O11	0.08354 (14)	0.15303 (9)	0.33467 (8)	0.0347 (3)	
O12	0.14141 (15)	−0.12645 (10)	0.58283 (10)	0.0436 (4)	
H12	0.0696	−0.1269	0.6056	0.065*	
O13	−0.01345 (13)	0.35115 (8)	0.20622 (9)	0.0344 (3)	
O14	−0.08956 (13)	0.24254 (9)	0.24632 (10)	0.0387 (4)	
O15	−0.28900 (18)	0.74434 (10)	0.22001 (11)	0.0515 (4)	
H15C	−0.3155	0.7677	0.2616	0.077*	
O16	0.25751 (14)	0.15768 (10)	0.07291 (9)	0.0373 (3)	
O17	0.06252 (16)	0.22753 (10)	0.08072 (10)	0.0456 (4)	
O18	0.3465 (2)	−0.21435 (11)	−0.05109 (14)	0.0692 (6)	
H18B	0.2901	−0.2320	−0.0546	0.104*	
H2WA	0.372 (3)	0.131 (2)	0.230 (2)	0.104*	
H2WB	0.355 (3)	0.0618 (12)	0.210 (2)	0.104*	
H3WB	0.496 (3)	0.749 (2)	0.497 (2)	0.104*	
H1WA	0.042 (3)	0.439 (2)	0.239 (2)	0.104*	
H1WB	0.068 (3)	0.5102 (12)	0.244 (2)	0.104*	
H3WA	0.597 (3)	0.734 (2)	0.519 (2)	0.104*	

### Atomic displacement parameters (Å^2^)

**Table:**

	*U*^11^	*U*^22^	*U*^33^	*U*^12^	*U*^13^	*U*^23^
Er1	0.02520 (4)	0.01535 (4)	0.03068 (5)	−0.00889 (3)	−0.01071 (3)	0.00290 (3)
Er2	0.02410 (4)	0.01427 (4)	0.03054 (5)	−0.00713 (3)	−0.00945 (3)	0.00068 (3)
N1	0.0306 (8)	0.0208 (8)	0.0434 (10)	−0.0104 (7)	−0.0122 (7)	0.0012 (7)
N2	0.0591 (14)	0.0278 (11)	0.121 (2)	−0.0221 (10)	−0.0450 (15)	0.0179 (13)
N3	0.0365 (9)	0.0195 (7)	0.0365 (10)	−0.0106 (7)	−0.0129 (7)	0.0011 (7)
N4	0.0824 (17)	0.0278 (11)	0.108 (2)	−0.0263 (11)	−0.0674 (17)	0.0176 (12)
C1	0.0371 (11)	0.0369 (11)	0.0299 (11)	−0.0138 (9)	−0.0115 (9)	−0.0023 (8)
C2	0.0367 (12)	0.0419 (12)	0.0407 (13)	−0.0099 (10)	−0.0026 (10)	−0.0119 (10)
C3	0.0320 (11)	0.0494 (14)	0.0520 (15)	−0.0164 (10)	−0.0013 (10)	−0.0079 (11)
C4	0.0374 (11)	0.0354 (11)	0.0514 (14)	−0.0144 (9)	−0.0139 (10)	−0.0033 (10)
C5	0.0369 (12)	0.0362 (12)	0.0557 (16)	−0.0063 (9)	−0.0061 (11)	−0.0140 (11)
C6	0.0320 (11)	0.0419 (12)	0.0481 (14)	−0.0139 (9)	−0.0031 (10)	−0.0074 (10)
C7	0.0499 (13)	0.0380 (12)	0.0318 (12)	−0.0201 (10)	−0.0083 (10)	−0.0002 (9)
C8	0.0425 (12)	0.0288 (10)	0.0336 (11)	−0.0220 (9)	−0.0077 (9)	0.0021 (8)
C9	0.0264 (10)	0.0244 (10)	0.0594 (15)	−0.0034 (8)	−0.0199 (10)	0.0001 (9)
C10	0.0695 (17)	0.0303 (11)	0.0508 (15)	−0.0085 (11)	−0.0286 (13)	−0.0083 (10)
C11	0.0694 (17)	0.0337 (12)	0.0367 (13)	−0.0096 (11)	−0.0169 (12)	−0.0015 (10)
C12	0.0425 (12)	0.0245 (10)	0.0411 (13)	−0.0103 (9)	−0.0117 (10)	−0.0006 (9)
C13	0.0631 (15)	0.0321 (11)	0.0367 (13)	−0.0145 (11)	−0.0132 (11)	−0.0063 (9)
C14	0.0523 (14)	0.0357 (12)	0.0389 (13)	−0.0115 (10)	−0.0128 (11)	0.0046 (10)
C15	0.0330 (11)	0.0256 (11)	0.090 (2)	−0.0076 (9)	−0.0287 (12)	0.0072 (11)
C16	0.0285 (10)	0.0234 (9)	0.0370 (11)	−0.0069 (7)	−0.0117 (8)	0.0022 (8)
C17	0.0663 (15)	0.0372 (12)	0.0267 (11)	−0.0289 (11)	−0.0184 (10)	0.0054 (9)
C18	0.0602 (15)	0.0567 (15)	0.0305 (12)	−0.0401 (13)	−0.0074 (11)	0.0022 (10)
C19	0.0403 (12)	0.0538 (14)	0.0392 (13)	−0.0193 (11)	−0.0101 (10)	−0.0070 (11)
C20	0.0471 (12)	0.0330 (11)	0.0370 (12)	−0.0171 (9)	−0.0183 (10)	0.0007 (9)
C21	0.0430 (12)	0.0360 (11)	0.0446 (13)	−0.0210 (10)	−0.0143 (10)	0.0058 (10)
C22	0.0459 (13)	0.0344 (11)	0.0442 (14)	−0.0161 (10)	−0.0188 (10)	0.0053 (10)
C23	0.104 (2)	0.0342 (12)	0.0367 (14)	−0.0352 (14)	−0.0248 (14)	0.0076 (10)
C24	0.0393 (11)	0.0229 (9)	0.0321 (11)	−0.0139 (8)	−0.0108 (9)	0.0031 (8)
C25	0.0526 (13)	0.0317 (10)	0.0319 (11)	−0.0227 (10)	−0.0208 (10)	0.0083 (9)
C26	0.0486 (13)	0.0309 (11)	0.0399 (13)	−0.0108 (10)	−0.0137 (10)	0.0034 (9)
C27	0.0436 (12)	0.0391 (12)	0.0363 (12)	−0.0180 (10)	−0.0080 (10)	0.0066 (9)
C28	0.0418 (11)	0.0309 (10)	0.0339 (11)	−0.0185 (9)	−0.0160 (9)	0.0081 (8)
C29	0.0395 (12)	0.0351 (11)	0.0418 (13)	−0.0117 (9)	−0.0111 (10)	0.0064 (9)
C30	0.0454 (12)	0.0420 (12)	0.0366 (12)	−0.0234 (10)	−0.0113 (10)	0.0116 (10)
C31	0.0680 (16)	0.0335 (11)	0.0382 (13)	−0.0293 (11)	−0.0256 (11)	0.0091 (9)
C32	0.0290 (9)	0.0227 (9)	0.0334 (11)	−0.0108 (7)	−0.0086 (8)	0.0041 (8)
C33	0.0240 (9)	0.0213 (9)	0.0385 (12)	−0.0033 (7)	−0.0103 (8)	−0.0030 (8)
C34	0.0373 (11)	0.0307 (10)	0.0344 (12)	−0.0057 (9)	−0.0072 (9)	−0.0091 (9)
C35	0.0454 (12)	0.0333 (11)	0.0329 (12)	−0.0127 (9)	−0.0082 (10)	0.0010 (9)
C36	0.0394 (11)	0.0228 (9)	0.0449 (13)	−0.0078 (8)	−0.0159 (10)	−0.0028 (9)
C37	0.0459 (12)	0.0285 (10)	0.0362 (12)	−0.0045 (9)	−0.0111 (10)	−0.0099 (9)
C38	0.0370 (11)	0.0299 (10)	0.0319 (11)	−0.0057 (8)	−0.0071 (9)	−0.0006 (8)
C39	0.0270 (10)	0.0221 (9)	0.0527 (14)	−0.0056 (8)	−0.0099 (9)	−0.0019 (9)
C40	0.0287 (9)	0.0220 (9)	0.0323 (11)	−0.0062 (7)	−0.0107 (8)	−0.0043 (7)
C41	0.0512 (13)	0.0356 (11)	0.0259 (11)	−0.0161 (10)	−0.0078 (9)	−0.0024 (8)
C42	0.0446 (12)	0.0386 (12)	0.0376 (13)	−0.0197 (10)	−0.0093 (10)	−0.0008 (9)
C43	0.0441 (13)	0.0390 (12)	0.0430 (14)	−0.0132 (10)	−0.0106 (10)	−0.0010 (10)
C44	0.0540 (14)	0.0350 (12)	0.0443 (14)	−0.0183 (11)	−0.0070 (11)	−0.0032 (10)
C45	0.0497 (14)	0.0477 (14)	0.0492 (15)	−0.0252 (11)	−0.0091 (11)	−0.0082 (11)
C46	0.0414 (12)	0.0448 (13)	0.0413 (14)	−0.0107 (10)	−0.0087 (10)	−0.0072 (10)
C47	0.0615 (15)	0.0347 (12)	0.0323 (12)	−0.0105 (11)	−0.0149 (11)	0.0006 (9)
C48	0.0486 (12)	0.0201 (9)	0.0331 (11)	−0.0142 (9)	−0.0106 (10)	0.0034 (8)
C49	0.0347 (11)	0.0275 (10)	0.0410 (12)	−0.0128 (8)	−0.0098 (9)	0.0013 (9)
C50	0.0397 (11)	0.0229 (10)	0.0441 (13)	−0.0091 (8)	−0.0129 (10)	−0.0016 (9)
C51	0.0366 (11)	0.0225 (9)	0.0494 (13)	−0.0121 (8)	−0.0209 (10)	0.0040 (9)
C52	0.0312 (11)	0.0279 (10)	0.0610 (16)	−0.0132 (9)	−0.0094 (10)	0.0039 (10)
C53	0.0313 (10)	0.0229 (10)	0.0580 (15)	−0.0091 (8)	−0.0086 (10)	−0.0031 (9)
C54	0.0471 (15)	0.0362 (15)	0.124 (3)	−0.0180 (12)	−0.0084 (17)	0.0222 (17)
C55	0.0439 (14)	0.0302 (12)	0.090 (2)	−0.0126 (10)	0.0005 (14)	0.0053 (13)
C56	0.0398 (12)	0.0246 (10)	0.0636 (16)	−0.0146 (9)	−0.0251 (11)	0.0071 (10)
C57	0.097 (2)	0.0343 (13)	0.0631 (18)	−0.0307 (14)	−0.0328 (16)	0.0016 (12)
C58	0.107 (3)	0.0350 (14)	0.087 (2)	−0.0307 (16)	−0.050 (2)	0.0001 (14)
C59	0.0442 (12)	0.0235 (10)	0.0394 (12)	−0.0129 (9)	−0.0085 (10)	0.0026 (8)
C60	0.0525 (13)	0.0221 (10)	0.0448 (14)	−0.0082 (9)	−0.0153 (11)	−0.0014 (9)
C61	0.0475 (12)	0.0211 (9)	0.0455 (13)	−0.0145 (9)	−0.0272 (10)	0.0055 (8)
C62	0.0373 (11)	0.0284 (10)	0.0585 (15)	−0.0168 (9)	−0.0160 (11)	0.0047 (10)
C63	0.0348 (11)	0.0227 (9)	0.0517 (14)	−0.0103 (8)	−0.0114 (10)	−0.0015 (9)
C64	0.125 (3)	0.0292 (12)	0.072 (2)	−0.0256 (15)	−0.062 (2)	0.0016 (13)
C65	0.110 (2)	0.0300 (12)	0.0532 (17)	−0.0282 (14)	−0.0406 (16)	0.0037 (11)
C66	0.0572 (14)	0.0236 (10)	0.0619 (16)	−0.0180 (10)	−0.0385 (13)	0.0077 (10)
C67	0.0432 (14)	0.0297 (12)	0.105 (2)	−0.0162 (10)	−0.0260 (15)	0.0120 (13)
C68	0.0514 (16)	0.0344 (14)	0.133 (3)	−0.0244 (12)	−0.0399 (18)	0.0235 (16)
O1W	0.0382 (8)	0.0189 (6)	0.0551 (10)	−0.0080 (6)	−0.0241 (7)	0.0009 (6)
O1	0.0510 (10)	0.0392 (9)	0.0437 (10)	−0.0072 (7)	−0.0170 (8)	−0.0072 (7)
O2W	0.0341 (8)	0.0186 (6)	0.0616 (11)	−0.0052 (6)	−0.0234 (7)	−0.0010 (7)
O2	0.0398 (8)	0.0425 (9)	0.0372 (9)	−0.0175 (7)	−0.0121 (7)	0.0004 (7)
O3	0.0497 (11)	0.0425 (10)	0.0940 (16)	−0.0228 (9)	−0.0057 (10)	−0.0149 (10)
O3W	0.0573 (12)	0.0537 (11)	0.0600 (12)	−0.0233 (9)	−0.0219 (10)	0.0168 (9)
O4	0.0323 (8)	0.0229 (7)	0.0730 (12)	−0.0109 (6)	−0.0184 (8)	0.0110 (7)
O5	0.0307 (7)	0.0217 (7)	0.0602 (10)	−0.0064 (6)	−0.0219 (7)	−0.0039 (6)
O6	0.0950 (15)	0.0282 (8)	0.0475 (11)	−0.0250 (9)	−0.0109 (10)	−0.0005 (7)
O7	0.0590 (10)	0.0458 (9)	0.0349 (9)	−0.0369 (8)	−0.0130 (7)	0.0053 (7)
O8	0.0317 (7)	0.0202 (6)	0.0348 (8)	−0.0119 (5)	−0.0072 (6)	0.0034 (5)
O9	0.0560 (10)	0.0330 (8)	0.0751 (13)	−0.0145 (8)	−0.0345 (10)	0.0077 (8)
O10	0.0310 (7)	0.0215 (6)	0.0324 (8)	−0.0133 (5)	−0.0086 (6)	0.0059 (5)
O11	0.0488 (9)	0.0330 (7)	0.0341 (8)	−0.0269 (7)	−0.0158 (7)	0.0076 (6)
O12	0.0485 (9)	0.0320 (8)	0.0500 (10)	−0.0185 (7)	−0.0114 (8)	0.0121 (7)
O13	0.0269 (7)	0.0196 (6)	0.0590 (10)	−0.0072 (5)	−0.0145 (7)	−0.0029 (6)
O14	0.0302 (7)	0.0196 (7)	0.0609 (11)	−0.0067 (6)	−0.0072 (7)	0.0028 (6)
O15	0.0759 (12)	0.0237 (8)	0.0580 (11)	−0.0152 (8)	−0.0232 (9)	−0.0001 (7)
O16	0.0394 (8)	0.0357 (8)	0.0404 (9)	−0.0148 (7)	−0.0116 (7)	−0.0034 (7)
O17	0.0472 (9)	0.0405 (9)	0.0424 (10)	0.0024 (7)	−0.0176 (8)	−0.0064 (7)
O18	0.0734 (13)	0.0327 (9)	0.1064 (18)	−0.0184 (9)	−0.0292 (12)	−0.0041 (10)

### Geometric parameters (Å, °)

**Table:**

Er1—O1W	2.3588 (14)	C28—O12	1.375 (2)
Er1—O5	2.3690 (13)	C28—C29	1.385 (3)
Er1—O10	2.3706 (12)	C29—C30	1.382 (3)
Er1—O4	2.3759 (14)	C29—H29	0.9300
Er1—O2	2.3842 (16)	C30—H30	0.9300
Er1—O7	2.3990 (15)	C31—C32	1.503 (3)
Er1—O1	2.5110 (17)	C31—H31A	0.9700
Er1—N1	2.5166 (15)	C31—H31B	0.9700
Er1—O8	2.5367 (14)	C32—O11	1.251 (2)
Er1—C16	2.7252 (19)	C32—O10	1.275 (2)
Er1—C8	2.823 (2)	C33—C38	1.382 (3)
Er1—C24	2.840 (2)	C33—C34	1.391 (3)
Er2—O8	2.3195 (12)	C33—C39	1.508 (3)
Er2—O14	2.3613 (14)	C34—C35	1.372 (3)
Er2—O2W	2.3630 (14)	C34—H34	0.9300
Er2—O13	2.3816 (13)	C35—C36	1.380 (3)
Er2—O16	2.3964 (15)	C35—H35	0.9300
Er2—O17	2.4762 (16)	C36—O15	1.364 (2)
Er2—O10	2.4986 (14)	C36—C37	1.383 (3)
Er2—O11	2.5271 (14)	C37—C38	1.387 (3)
Er2—N3	2.5365 (15)	C37—H37	0.9300
Er2—C40	2.7432 (19)	C38—H38	0.9300
Er2—C48	2.815 (2)	C39—C40	1.508 (3)
Er2—C32	2.892 (2)	C39—H39A	0.9700
N1—C49	1.336 (3)	C39—H39B	0.9700
N1—C53	1.340 (3)	C40—O14	1.256 (2)
N2—C58	1.316 (4)	C40—O13	1.262 (2)
N2—C54	1.319 (4)	C41—C46	1.384 (3)
N3—C59	1.333 (3)	C41—C42	1.391 (3)
N3—C63	1.341 (3)	C41—C47	1.514 (3)
N4—C64	1.323 (4)	C42—C43	1.378 (3)
N4—C68	1.328 (4)	C42—H42	0.9300
C1—C2	1.383 (3)	C43—C44	1.381 (3)
C1—C6	1.386 (3)	C43—H43	0.9300
C1—C7	1.518 (3)	C44—O18	1.364 (3)
C2—C3	1.383 (3)	C44—C45	1.383 (4)
C2—H2	0.9300	C45—C46	1.382 (3)
C3—C4	1.374 (3)	C45—H45	0.9300
C3—H3	0.9300	C46—H46	0.9300
C4—O3	1.375 (3)	C47—C48	1.511 (3)
C4—C5	1.375 (3)	C47—H47A	0.9700
C5—C6	1.380 (3)	C47—H47B	0.9700
C5—H5	0.9300	C48—O16	1.252 (3)
C6—H6	0.9300	C48—O17	1.263 (3)
C7—C8	1.506 (3)	C49—C50	1.384 (3)
C7—H7A	0.9700	C49—H49	0.9300
C7—H7B	0.9700	C50—C51	1.387 (3)
C8—O1	1.256 (3)	C50—H50	0.9300
C8—O2	1.258 (3)	C51—C52	1.386 (3)
C9—C10	1.373 (3)	C51—C56	1.485 (3)
C9—C14	1.385 (3)	C52—C53	1.378 (3)
C9—C15	1.505 (3)	C52—H52	0.9300
C10—C11	1.386 (3)	C53—H53	0.9300
C10—H10	0.9300	C54—C55	1.383 (3)
C11—C12	1.375 (3)	C54—H54	0.9300
C11—H11	0.9300	C55—C56	1.380 (4)
C12—O6	1.371 (2)	C55—H55	0.9300
C12—C13	1.373 (3)	C56—C57	1.377 (4)
C13—C14	1.375 (3)	C57—C58	1.385 (4)
C13—H13	0.9300	C57—H57	0.9300
C14—H14	0.9300	C58—H58	0.9300
C15—C16	1.509 (3)	C59—C60	1.386 (3)
C15—H15A	0.9700	C59—H59	0.9300
C15—H15B	0.9700	C60—C61	1.378 (3)
C16—O4	1.246 (2)	C60—H60	0.9300
C16—O5	1.265 (2)	C61—C62	1.387 (3)
C17—C18	1.384 (4)	C61—C66	1.490 (3)
C17—C22	1.385 (3)	C62—C63	1.379 (3)
C17—C23	1.511 (3)	C62—H62	0.9300
C18—C19	1.389 (3)	C63—H63	0.9300
C18—H18	0.9300	C64—C65	1.386 (3)
C19—C20	1.385 (3)	C64—H64	0.9300
C19—H19	0.9300	C65—C66	1.387 (4)
C20—O9	1.371 (3)	C65—H65	0.9300
C20—C21	1.380 (3)	C66—C67	1.385 (4)
C21—C22	1.381 (3)	C67—C68	1.384 (3)
C21—H21	0.9300	C67—H67	0.9300
C22—H22	0.9300	C68—H68	0.9300
C23—C24	1.506 (3)	O1W—H1WA	0.83 (4)
C23—H23A	0.9700	O1W—H1WB	0.830 (17)
C23—H23B	0.9700	O2W—H2WA	0.83 (4)
C24—O7	1.243 (2)	O2W—H2WB	0.812 (17)
C24—O8	1.268 (2)	O3—H3B	0.8200
C25—C30	1.380 (3)	O3W—H3WB	0.82 (4)
C25—C26	1.385 (3)	O3W—H3WA	0.84 (4)
C25—C31	1.506 (3)	O6—H6B	0.8200
C26—C27	1.391 (3)	O9—H9A	0.8200
C26—H26	0.9300	O12—H12	0.8200
C27—C28	1.373 (3)	O15—H15C	0.8200
C27—H27	0.9300	O18—H18B	0.8200
			
O1W—Er1—O5	145.68 (5)	C19—C18—H18	119.3
O1W—Er1—O10	79.74 (5)	C20—C19—C18	119.6 (2)
O5—Er1—O10	73.55 (5)	C20—C19—H19	120.2
O1W—Er1—O4	149.18 (5)	C18—C19—H19	120.2
O5—Er1—O4	54.63 (5)	O9—C20—C21	118.1 (2)
O10—Er1—O4	127.84 (5)	O9—C20—C19	122.3 (2)
O1W—Er1—O2	74.64 (6)	C21—C20—C19	119.6 (2)
O5—Er1—O2	122.21 (5)	C20—C21—C22	120.1 (2)
O10—Er1—O2	83.66 (5)	C20—C21—H21	119.9
O4—Er1—O2	117.27 (6)	C22—C21—H21	119.9
O1W—Er1—O7	77.95 (6)	C21—C22—C17	121.3 (2)
O5—Er1—O7	95.38 (6)	C21—C22—H22	119.3
O10—Er1—O7	117.22 (5)	C17—C22—H22	119.3
O4—Er1—O7	76.65 (6)	C24—C23—C17	112.22 (19)
O2—Er1—O7	141.64 (5)	C24—C23—H23A	109.2
O1W—Er1—O1	127.08 (6)	C17—C23—H23A	109.2
O5—Er1—O1	75.31 (5)	C24—C23—H23B	109.2
O10—Er1—O1	91.26 (5)	C17—C23—H23B	109.2
O4—Er1—O1	71.84 (6)	H23A—C23—H23B	107.9
O2—Er1—O1	52.49 (5)	O7—C24—O8	119.71 (19)
O7—Er1—O1	146.60 (5)	O7—C24—C23	120.06 (18)
O1W—Er1—N1	80.79 (5)	O8—C24—C23	120.22 (18)
O5—Er1—N1	130.56 (5)	O7—C24—Er1	56.90 (11)
O10—Er1—N1	153.76 (5)	O8—C24—Er1	63.28 (11)
O4—Er1—N1	76.11 (5)	C23—C24—Er1	171.85 (16)
O2—Er1—N1	74.32 (5)	C30—C25—C26	118.20 (19)
O7—Er1—N1	75.24 (5)	C30—C25—C31	119.9 (2)
O1—Er1—N1	86.59 (6)	C26—C25—C31	121.9 (2)
O1W—Er1—O8	73.77 (5)	C25—C26—C27	121.1 (2)
O5—Er1—O8	75.72 (5)	C25—C26—H26	119.5
O10—Er1—O8	65.45 (4)	C27—C26—H26	119.5
O4—Er1—O8	103.26 (5)	C28—C27—C26	119.7 (2)
O2—Er1—O8	138.92 (5)	C28—C27—H27	120.2
O7—Er1—O8	52.10 (4)	C26—C27—H27	120.2
O1—Er1—O8	146.97 (5)	C27—C28—O12	122.33 (19)
N1—Er1—O8	124.76 (5)	C27—C28—C29	120.08 (19)
O1W—Er1—C16	163.82 (6)	O12—C28—C29	117.6 (2)
O5—Er1—C16	27.64 (5)	C30—C29—C28	119.6 (2)
O10—Er1—C16	100.67 (5)	C30—C29—H29	120.2
O4—Er1—C16	27.18 (5)	C28—C29—H29	120.2
O2—Er1—C16	121.53 (6)	C25—C30—C29	121.4 (2)
O7—Er1—C16	87.70 (6)	C25—C30—H30	119.3
O1—Er1—C16	69.08 (6)	C29—C30—H30	119.3
N1—Er1—C16	102.92 (5)	C32—C31—C25	114.86 (17)
O8—Er1—C16	91.58 (5)	C32—C31—H31A	108.6
O1W—Er1—C8	100.87 (6)	C25—C31—H31A	108.6
O5—Er1—C8	100.00 (6)	C32—C31—H31B	108.6
O10—Er1—C8	89.38 (5)	C25—C31—H31B	108.6
O4—Er1—C8	93.65 (6)	H31A—C31—H31B	107.5
O2—Er1—C8	26.26 (6)	O11—C32—O10	119.11 (19)
O7—Er1—C8	152.25 (5)	O11—C32—C31	122.79 (17)
O1—Er1—C8	26.41 (6)	O10—C32—C31	118.09 (17)
N1—Er1—C8	77.21 (6)	O11—C32—Er2	60.70 (11)
O8—Er1—C8	154.75 (5)	O10—C32—Er2	59.49 (10)
C16—Er1—C8	95.30 (6)	C31—C32—Er2	169.24 (15)
O1W—Er1—C24	72.34 (6)	C38—C33—C34	117.80 (18)
O5—Er1—C24	87.05 (6)	C38—C33—C39	121.43 (19)
O10—Er1—C24	91.52 (5)	C34—C33—C39	120.77 (19)
O4—Er1—C24	91.39 (6)	C35—C34—C33	121.5 (2)
O2—Er1—C24	146.96 (6)	C35—C34—H34	119.3
O7—Er1—C24	25.73 (5)	C33—C34—H34	119.3
O1—Er1—C24	160.55 (6)	C34—C35—C36	120.2 (2)
N1—Er1—C24	99.15 (6)	C34—C35—H35	119.9
O8—Er1—C24	26.51 (5)	C36—C35—H35	119.9
C16—Er1—C24	91.50 (6)	O15—C36—C35	117.8 (2)
C8—Er1—C24	172.85 (6)	O15—C36—C37	122.7 (2)
O8—Er2—O14	129.11 (5)	C35—C36—C37	119.51 (19)
O8—Er2—O2W	78.64 (5)	C36—C37—C38	119.8 (2)
O14—Er2—O2W	143.03 (6)	C36—C37—H37	120.1
O8—Er2—O13	75.23 (5)	C38—C37—H37	120.1
O14—Er2—O13	54.55 (5)	C33—C38—C37	121.3 (2)
O2W—Er2—O13	148.14 (5)	C33—C38—H38	119.4
O8—Er2—O16	80.14 (5)	C37—C38—H38	119.4
O14—Er2—O16	127.19 (5)	C40—C39—C33	115.33 (16)
O2W—Er2—O16	75.46 (6)	C40—C39—H39A	108.4
O13—Er2—O16	116.92 (5)	C33—C39—H39A	108.4
O8—Er2—O17	99.32 (5)	C40—C39—H39B	108.4
O14—Er2—O17	77.25 (6)	C33—C39—H39B	108.4
O2W—Er2—O17	127.31 (6)	H39A—C39—H39B	107.5
O13—Er2—O17	75.31 (5)	O14—C40—O13	119.36 (17)
O16—Er2—O17	52.78 (5)	O14—C40—C39	118.66 (17)
O8—Er2—O10	66.80 (5)	O13—C40—C39	121.96 (17)
O14—Er2—O10	91.38 (5)	O14—C40—Er2	59.22 (10)
O2W—Er2—O10	77.23 (5)	O13—C40—Er2	60.15 (9)
O13—Er2—O10	75.92 (5)	C39—C40—Er2	176.75 (14)
O16—Er2—O10	140.46 (5)	C46—C41—C42	117.2 (2)
O17—Er2—O10	150.48 (5)	C46—C41—C47	120.8 (2)
O8—Er2—O11	115.63 (5)	C42—C41—C47	122.0 (2)
O14—Er2—O11	72.76 (5)	C43—C42—C41	122.0 (2)
O2W—Er2—O11	72.76 (6)	C43—C42—H42	119.0
O13—Er2—O11	102.66 (5)	C41—C42—H42	119.0
O16—Er2—O11	140.23 (5)	C42—C43—C44	119.6 (2)
O17—Er2—O11	143.49 (5)	C42—C43—H43	120.2
O10—Er2—O11	51.35 (4)	C44—C43—H43	120.2
O8—Er2—N3	153.52 (5)	O18—C44—C43	117.6 (2)
O14—Er2—N3	76.20 (5)	O18—C44—C45	122.7 (2)
O2W—Er2—N3	83.00 (5)	C43—C44—C45	119.7 (2)
O13—Er2—N3	127.36 (5)	C46—C45—C44	119.9 (2)
O16—Er2—N3	76.87 (5)	C46—C45—H45	120.1
O17—Er2—N3	76.94 (6)	C44—C45—H45	120.1
O10—Er2—N3	127.16 (5)	C45—C46—C41	121.7 (2)
O11—Er2—N3	76.10 (5)	C45—C46—H46	119.2
O8—Er2—C40	102.34 (5)	C41—C46—H46	119.2
O14—Er2—C40	27.19 (5)	C48—C47—C41	111.65 (18)
O2W—Er2—C40	158.33 (6)	C48—C47—H47A	109.3
O13—Er2—C40	27.36 (5)	C41—C47—H47A	109.3
O16—Er2—C40	126.18 (6)	C48—C47—H47B	109.3
O17—Er2—C40	74.19 (6)	C41—C47—H47B	109.3
O10—Er2—C40	83.26 (5)	H47A—C47—H47B	108.0
O11—Er2—C40	87.74 (5)	O16—C48—O17	119.0 (2)
N3—Er2—C40	101.76 (5)	O16—C48—C47	120.3 (2)
O8—Er2—C48	91.40 (5)	O17—C48—C47	120.7 (2)
O14—Er2—C48	101.95 (6)	O16—C48—Er2	57.88 (11)
O2W—Er2—C48	100.90 (6)	O17—C48—Er2	61.55 (11)
O13—Er2—C48	97.68 (6)	C47—C48—Er2	172.10 (14)
O16—Er2—C48	26.26 (6)	N1—C49—C50	123.4 (2)
O17—Er2—C48	26.64 (6)	N1—C49—H49	118.3
O10—Er2—C48	158.14 (5)	C50—C49—H49	118.3
O11—Er2—C48	149.45 (5)	C49—C50—C51	119.5 (2)
N3—Er2—C48	73.45 (6)	C49—C50—H50	120.3
C40—Er2—C48	100.72 (6)	C51—C50—H50	120.3
O8—Er2—C32	90.60 (5)	C52—C51—C50	117.21 (18)
O14—Er2—C32	83.90 (6)	C52—C51—C56	121.8 (2)
O2W—Er2—C32	70.41 (6)	C50—C51—C56	121.0 (2)
O13—Er2—C32	91.82 (5)	C53—C52—C51	119.7 (2)
O16—Er2—C32	145.78 (5)	C53—C52—H52	120.1
O17—Er2—C32	160.99 (5)	C51—C52—H52	120.1
O10—Er2—C32	26.08 (5)	N1—C53—C52	123.4 (2)
O11—Er2—C32	25.57 (5)	N1—C53—H53	118.3
N3—Er2—C32	101.08 (5)	C52—C53—H53	118.3
C40—Er2—C32	87.92 (6)	N2—C54—C55	124.7 (3)
C48—Er2—C32	170.50 (6)	N2—C54—H54	117.7
C49—N1—C53	116.82 (17)	C55—C54—H54	117.7
C49—N1—Er1	123.99 (13)	C56—C55—C54	119.0 (3)
C53—N1—Er1	118.96 (13)	C56—C55—H55	120.5
C58—N2—C54	115.9 (2)	C54—C55—H55	120.5
C59—N3—C63	116.50 (17)	C57—C56—C55	116.7 (2)
C59—N3—Er2	121.24 (13)	C57—C56—C51	122.2 (2)
C63—N3—Er2	122.07 (13)	C55—C56—C51	121.1 (2)
C64—N4—C68	116.3 (2)	C56—C57—C58	119.6 (3)
C2—C1—C6	117.8 (2)	C56—C57—H57	120.2
C2—C1—C7	122.2 (2)	C58—C57—H57	120.2
C6—C1—C7	119.97 (19)	N2—C58—C57	124.1 (3)
C3—C2—C1	121.1 (2)	N2—C58—H58	118.0
C3—C2—H2	119.4	C57—C58—H58	118.0
C1—C2—H2	119.4	N3—C59—C60	123.2 (2)
C4—C3—C2	120.1 (2)	N3—C59—H59	118.4
C4—C3—H3	119.9	C60—C59—H59	118.4
C2—C3—H3	119.9	C61—C60—C59	120.3 (2)
C3—C4—O3	118.6 (2)	C61—C60—H60	119.9
C3—C4—C5	119.7 (2)	C59—C60—H60	119.9
O3—C4—C5	121.7 (2)	C60—C61—C62	116.39 (18)
C4—C5—C6	119.9 (2)	C60—C61—C66	120.9 (2)
C4—C5—H5	120.1	C62—C61—C66	122.6 (2)
C6—C5—H5	120.1	C63—C62—C61	120.0 (2)
C5—C6—C1	121.4 (2)	C63—C62—H62	120.0
C5—C6—H6	119.3	C61—C62—H62	120.0
C1—C6—H6	119.3	N3—C63—C62	123.4 (2)
C8—C7—C1	111.32 (17)	N3—C63—H63	118.3
C8—C7—H7A	109.4	C62—C63—H63	118.3
C1—C7—H7A	109.4	N4—C64—C65	123.9 (3)
C8—C7—H7B	109.4	N4—C64—H64	118.1
C1—C7—H7B	109.4	C65—C64—H64	118.1
H7A—C7—H7B	108.0	C64—C65—C66	119.5 (3)
O1—C8—O2	119.2 (2)	C64—C65—H65	120.3
O1—C8—C7	121.0 (2)	C66—C65—H65	120.3
O2—C8—C7	119.8 (2)	C67—C66—C65	116.8 (2)
O1—C8—Er1	62.80 (12)	C67—C66—C61	121.1 (2)
O2—C8—Er1	57.01 (11)	C65—C66—C61	122.0 (2)
C7—C8—Er1	169.72 (14)	C68—C67—C66	119.1 (3)
C10—C9—C14	117.3 (2)	C68—C67—H67	120.5
C10—C9—C15	121.9 (2)	C66—C67—H67	120.5
C14—C9—C15	120.8 (2)	N4—C68—C67	124.3 (3)
C9—C10—C11	122.2 (2)	N4—C68—H68	117.8
C9—C10—H10	118.9	C67—C68—H68	117.8
C11—C10—H10	118.9	Er1—O1W—H1WA	123 (2)
C12—C11—C10	119.1 (2)	Er1—O1W—H1WB	132 (2)
C12—C11—H11	120.4	H1WA—O1W—H1WB	103 (2)
C10—C11—H11	120.4	C8—O1—Er1	90.79 (13)
O6—C12—C13	117.7 (2)	Er2—O2W—H2WA	123 (2)
O6—C12—C11	122.5 (2)	Er2—O2W—H2WB	131 (2)
C13—C12—C11	119.8 (2)	H2WA—O2W—H2WB	105 (2)
C12—C13—C14	120.2 (2)	C8—O2—Er1	96.74 (13)
C12—C13—H13	119.9	C4—O3—H3B	109.5
C14—C13—H13	119.9	H3WB—O3W—H3WA	105 (2)
C13—C14—C9	121.4 (2)	C16—O4—Er1	92.22 (12)
C13—C14—H14	119.3	C16—O5—Er1	92.05 (11)
C9—C14—H14	119.3	C12—O6—H6B	109.5
C9—C15—C16	115.35 (18)	C24—O7—Er1	97.37 (12)
C9—C15—H15A	108.4	C24—O8—Er2	153.46 (14)
C16—C15—H15A	108.4	C24—O8—Er1	90.21 (12)
C9—C15—H15B	108.4	Er2—O8—Er1	114.09 (5)
C16—C15—H15B	108.4	C20—O9—H9A	109.5
H15A—C15—H15B	107.5	C32—O10—Er1	142.26 (13)
O4—C16—O5	120.27 (18)	C32—O10—Er2	94.43 (12)
O4—C16—C15	119.32 (18)	Er1—O10—Er2	113.66 (5)
O5—C16—C15	120.38 (17)	C32—O11—Er2	93.72 (12)
O4—C16—Er1	60.59 (10)	C28—O12—H12	109.5
O5—C16—Er1	60.31 (10)	C40—O13—Er2	92.48 (11)
C15—C16—Er1	170.25 (17)	C40—O14—Er2	93.59 (11)
C18—C17—C22	118.0 (2)	C36—O15—H15C	109.5
C18—C17—C23	120.9 (2)	C48—O16—Er2	95.86 (13)
C22—C17—C23	121.1 (2)	C48—O17—Er2	91.80 (13)
C17—C18—C19	121.4 (2)	C44—O18—H18B	109.5
C17—C18—H18	119.3		
			
O1W—Er1—N1—C49	51.42 (17)	C40—Er2—C48—O17	−5.39 (13)
O5—Er1—N1—C49	−144.33 (15)	C53—N1—C49—C50	1.6 (3)
O10—Er1—N1—C49	8.9 (2)	Er1—N1—C49—C50	−172.94 (16)
O4—Er1—N1—C49	−149.13 (18)	N1—C49—C50—C51	0.4 (3)
O2—Er1—N1—C49	−25.09 (16)	C49—C50—C51—C52	−2.0 (3)
O7—Er1—N1—C49	131.28 (17)	C49—C50—C51—C56	176.0 (2)
O1—Er1—N1—C49	−77.00 (17)	C50—C51—C52—C53	1.6 (3)
O8—Er1—N1—C49	114.21 (16)	C56—C51—C52—C53	−176.3 (2)
C16—Er1—N1—C49	−144.62 (17)	C49—N1—C53—C52	−1.9 (3)
C8—Er1—N1—C49	−52.03 (17)	Er1—N1—C53—C52	172.85 (18)
C24—Er1—N1—C49	121.70 (17)	C51—C52—C53—N1	0.4 (4)
O1W—Er1—N1—C53	−122.96 (17)	C58—N2—C54—C55	2.1 (5)
O5—Er1—N1—C53	41.28 (19)	N2—C54—C55—C56	−1.1 (5)
O10—Er1—N1—C53	−165.47 (14)	C54—C55—C56—C57	−1.3 (4)
O4—Er1—N1—C53	36.48 (16)	C54—C55—C56—C51	175.8 (2)
O2—Er1—N1—C53	160.52 (17)	C52—C51—C56—C57	−154.9 (3)
O7—Er1—N1—C53	−43.10 (16)	C50—C51—C56—C57	27.2 (3)
O1—Er1—N1—C53	108.61 (16)	C52—C51—C56—C55	28.1 (3)
O8—Er1—N1—C53	−60.17 (17)	C50—C51—C56—C55	−149.8 (2)
C16—Er1—N1—C53	40.99 (17)	C55—C56—C57—C58	2.7 (4)
C8—Er1—N1—C53	133.58 (17)	C51—C56—C57—C58	−174.4 (3)
C24—Er1—N1—C53	−52.69 (17)	C54—N2—C58—C57	−0.5 (5)
O8—Er2—N3—C59	0.2 (2)	C56—C57—C58—N2	−1.9 (5)
O14—Er2—N3—C59	164.69 (17)	C63—N3—C59—C60	−2.1 (3)
O2W—Er2—N3—C59	−46.10 (16)	Er2—N3—C59—C60	172.80 (17)
O13—Er2—N3—C59	144.55 (15)	N3—C59—C60—C61	−1.6 (4)
O16—Er2—N3—C59	30.55 (16)	C59—C60—C61—C62	4.1 (3)
O17—Er2—N3—C59	84.85 (16)	C59—C60—C61—C66	−173.2 (2)
O10—Er2—N3—C59	−114.23 (16)	C60—C61—C62—C63	−3.0 (3)
O11—Er2—N3—C59	−120.00 (16)	C66—C61—C62—C63	174.3 (2)
C40—Er2—N3—C59	155.34 (16)	C59—N3—C63—C62	3.4 (3)
C48—Er2—N3—C59	57.51 (16)	Er2—N3—C63—C62	−171.53 (17)
C32—Er2—N3—C59	−114.49 (16)	C61—C62—C63—N3	−0.8 (4)
O8—Er2—N3—C63	174.81 (14)	C68—N4—C64—C65	0.7 (4)
O14—Er2—N3—C63	−20.66 (16)	N4—C64—C65—C66	1.4 (4)
O2W—Er2—N3—C63	128.56 (17)	C64—C65—C66—C67	−2.1 (4)
O13—Er2—N3—C63	−40.79 (19)	C64—C65—C66—C61	174.5 (2)
O16—Er2—N3—C63	−154.80 (17)	C60—C61—C66—C67	159.6 (2)
O17—Er2—N3—C63	−100.50 (17)	C62—C61—C66—C67	−17.6 (3)
O10—Er2—N3—C63	60.43 (18)	C60—C61—C66—C65	−16.8 (3)
O11—Er2—N3—C63	54.66 (16)	C62—C61—C66—C65	166.0 (2)
C40—Er2—N3—C63	−30.00 (17)	C65—C66—C67—C68	0.9 (4)
C48—Er2—N3—C63	−127.84 (17)	C61—C66—C67—C68	−175.7 (2)
C32—Er2—N3—C63	60.16 (17)	C64—N4—C68—C67	−2.0 (4)
C6—C1—C2—C3	−1.1 (4)	C66—C67—C68—N4	1.2 (5)
C7—C1—C2—C3	−179.4 (2)	O2—C8—O1—Er1	8.74 (19)
C1—C2—C3—C4	0.6 (4)	C7—C8—O1—Er1	−169.05 (16)
C2—C3—C4—O3	−179.8 (2)	O1W—Er1—O1—C8	−8.01 (14)
C2—C3—C4—C5	1.0 (4)	O5—Er1—O1—C8	−158.70 (13)
O3—C4—C5—C6	178.8 (2)	O10—Er1—O1—C8	−86.08 (12)
C3—C4—C5—C6	−1.9 (4)	O4—Er1—O1—C8	144.24 (13)
C4—C5—C6—C1	1.4 (4)	O2—Er1—O1—C8	−5.06 (11)
C2—C1—C6—C5	0.1 (4)	O7—Er1—O1—C8	124.10 (13)
C7—C1—C6—C5	178.5 (2)	N1—Er1—O1—C8	67.75 (12)
C2—C1—C7—C8	−47.9 (3)	O8—Er1—O1—C8	−129.30 (12)
C6—C1—C7—C8	133.8 (2)	C16—Er1—O1—C8	172.97 (13)
C1—C7—C8—O1	96.6 (2)	C24—Er1—O1—C8	175.78 (14)
C1—C7—C8—O2	−81.2 (2)	O1—C8—O2—Er1	−9.3 (2)
C1—C7—C8—Er1	−12.1 (10)	C7—C8—O2—Er1	168.54 (15)
O1W—Er1—C8—O1	173.50 (12)	O1W—Er1—O2—C8	−177.35 (12)
O5—Er1—C8—O1	20.91 (13)	O5—Er1—O2—C8	35.60 (13)
O10—Er1—C8—O1	94.08 (12)	O10—Er1—O2—C8	101.59 (12)
O4—Er1—C8—O1	−33.81 (12)	O4—Er1—O2—C8	−27.99 (13)
O2—Er1—C8—O1	170.90 (19)	O7—Er1—O2—C8	−131.46 (12)
O7—Er1—C8—O1	−101.76 (16)	O1—Er1—O2—C8	5.09 (11)
N1—Er1—C8—O1	−108.66 (12)	N1—Er1—O2—C8	−92.81 (12)
O8—Er1—C8—O1	98.58 (16)	O8—Er1—O2—C8	141.80 (11)
C16—Er1—C8—O1	−6.59 (13)	C16—Er1—O2—C8	2.94 (14)
O1W—Er1—C8—O2	2.60 (12)	C24—Er1—O2—C8	−175.42 (11)
O5—Er1—C8—O2	−149.99 (11)	O5—C16—O4—Er1	9.2 (2)
O10—Er1—C8—O2	−76.82 (12)	C15—C16—O4—Er1	−168.8 (2)
O4—Er1—C8—O2	155.30 (12)	O1W—Er1—O4—C16	−147.08 (13)
O7—Er1—C8—O2	87.34 (17)	O5—Er1—O4—C16	−5.18 (12)
O1—Er1—C8—O2	−170.90 (19)	O10—Er1—O4—C16	2.44 (16)
N1—Er1—C8—O2	80.44 (12)	O2—Er1—O4—C16	106.51 (14)
O8—Er1—C8—O2	−72.31 (17)	O7—Er1—O4—C16	−111.83 (14)
C16—Er1—C8—O2	−177.49 (12)	O1—Er1—O4—C16	79.40 (14)
O1W—Er1—C8—C7	−72.5 (9)	N1—Er1—O4—C16	170.35 (14)
O5—Er1—C8—C7	134.9 (9)	O8—Er1—O4—C16	−66.62 (14)
O10—Er1—C8—C7	−151.9 (9)	C8—Er1—O4—C16	94.50 (14)
O4—Er1—C8—C7	80.2 (9)	C24—Er1—O4—C16	−90.57 (14)
O2—Er1—C8—C7	−75.1 (9)	O4—C16—O5—Er1	−9.2 (2)
O7—Er1—C8—C7	12.3 (10)	C15—C16—O5—Er1	168.8 (2)
O1—Er1—C8—C7	114.0 (9)	O1W—Er1—O5—C16	151.00 (13)
N1—Er1—C8—C7	5.4 (9)	O10—Er1—O5—C16	−168.64 (14)
O8—Er1—C8—C7	−147.4 (8)	O4—Er1—O5—C16	5.10 (12)
C16—Er1—C8—C7	107.4 (9)	O2—Er1—O5—C16	−97.44 (13)
C14—C9—C10—C11	0.8 (4)	O7—Er1—O5—C16	74.54 (13)
C15—C9—C10—C11	−179.1 (2)	O1—Er1—O5—C16	−72.83 (13)
C9—C10—C11—C12	0.2 (4)	N1—Er1—O5—C16	−0.62 (16)
C10—C11—C12—O6	178.7 (2)	O8—Er1—O5—C16	123.19 (13)
C10—C11—C12—C13	−0.9 (4)	C8—Er1—O5—C16	−82.28 (13)
O6—C12—C13—C14	−178.9 (2)	C24—Er1—O5—C16	98.90 (13)
C11—C12—C13—C14	0.7 (4)	O8—C24—O7—Er1	−8.1 (2)
C12—C13—C14—C9	0.3 (4)	C23—C24—O7—Er1	171.12 (19)
C10—C9—C14—C13	−1.0 (4)	O1W—Er1—O7—C24	−74.24 (13)
C15—C9—C14—C13	178.9 (2)	O5—Er1—O7—C24	71.67 (13)
C10—C9—C15—C16	−97.4 (3)	O10—Er1—O7—C24	−2.58 (15)
C14—C9—C15—C16	82.8 (3)	O4—Er1—O7—C24	123.36 (14)
C9—C15—C16—O4	172.5 (2)	O2—Er1—O7—C24	−119.31 (13)
C9—C15—C16—O5	−5.5 (4)	O1—Er1—O7—C24	143.00 (13)
O1W—Er1—C16—O4	92.0 (2)	N1—Er1—O7—C24	−157.75 (14)
O5—Er1—C16—O4	170.9 (2)	O8—Er1—O7—C24	4.45 (12)
O10—Er1—C16—O4	−178.04 (13)	C16—Er1—O7—C24	98.25 (14)
O2—Er1—C16—O4	−88.97 (14)	C8—Er1—O7—C24	−164.72 (14)
O7—Er1—C16—O4	64.68 (14)	O7—C24—O8—Er2	164.66 (19)
O1—Er1—C16—O4	−90.80 (14)	C23—C24—O8—Er2	−14.6 (4)
N1—Er1—C16—O4	−9.61 (14)	Er1—C24—O8—Er2	157.0 (3)
O8—Er1—C16—O4	116.65 (13)	O7—C24—O8—Er1	7.6 (2)
C8—Er1—C16—O4	−87.67 (14)	C23—C24—O8—Er1	−171.6 (2)
C24—Er1—C16—O4	90.13 (14)	O14—Er2—O8—C24	−82.9 (3)
O1W—Er1—C16—O5	−78.9 (2)	O2W—Er2—O8—C24	124.6 (3)
O10—Er1—C16—O5	11.08 (13)	O13—Er2—O8—C24	−73.8 (3)
O4—Er1—C16—O5	−170.9 (2)	O16—Er2—O8—C24	47.6 (3)
O2—Er1—C16—O5	100.15 (13)	O17—Er2—O8—C24	−1.8 (3)
O7—Er1—C16—O5	−106.19 (13)	O10—Er2—O8—C24	−154.5 (3)
O1—Er1—C16—O5	98.32 (13)	O11—Er2—O8—C24	−170.9 (3)
N1—Er1—C16—O5	179.52 (12)	N3—Er2—O8—C24	77.6 (3)
O8—Er1—C16—O5	−54.22 (13)	C40—Er2—O8—C24	−77.5 (3)
C8—Er1—C16—O5	101.46 (13)	C48—Er2—O8—C24	23.8 (3)
C24—Er1—C16—O5	−80.74 (13)	C32—Er2—O8—C24	−165.5 (3)
C22—C17—C18—C19	0.7 (3)	O14—Er2—O8—Er1	71.85 (8)
C23—C17—C18—C19	179.3 (2)	O2W—Er2—O8—Er1	−80.65 (6)
C17—C18—C19—C20	0.1 (4)	O13—Er2—O8—Er1	80.95 (6)
C18—C19—C20—O9	180.0 (2)	O16—Er2—O8—Er1	−157.65 (6)
C18—C19—C20—C21	−1.0 (3)	O17—Er2—O8—Er1	152.95 (6)
O9—C20—C21—C22	−180.0 (2)	O10—Er2—O8—Er1	0.20 (4)
C19—C20—C21—C22	1.0 (3)	O11—Er2—O8—Er1	−16.22 (7)
C20—C21—C22—C17	−0.1 (4)	N3—Er2—O8—Er1	−127.64 (10)
C18—C17—C22—C21	−0.7 (3)	C40—Er2—O8—Er1	77.23 (6)
C23—C17—C22—C21	−179.3 (2)	C48—Er2—O8—Er1	178.52 (6)
C18—C17—C23—C24	−78.7 (3)	C32—Er2—O8—Er1	−10.77 (6)
C22—C17—C23—C24	99.8 (3)	O1W—Er1—O8—C24	82.86 (12)
C17—C23—C24—O7	−46.4 (3)	O5—Er1—O8—C24	−113.04 (12)
C17—C23—C24—O8	132.8 (2)	O10—Er1—O8—C24	168.79 (13)
O1W—Er1—C24—O7	98.98 (14)	O4—Er1—O8—C24	−65.39 (12)
O5—Er1—C24—O7	−108.86 (13)	O2—Er1—O8—C24	123.93 (12)
O10—Er1—C24—O7	177.70 (13)	O7—Er1—O8—C24	−4.33 (11)
O4—Er1—C24—O7	−54.38 (13)	O1—Er1—O8—C24	−142.38 (12)
O2—Er1—C24—O7	97.03 (16)	N1—Er1—O8—C24	16.75 (13)
O1—Er1—C24—O7	−84.2 (2)	C16—Er1—O8—C24	−90.19 (12)
N1—Er1—C24—O7	21.77 (14)	C8—Er1—O8—C24	163.83 (14)
O8—Er1—C24—O7	−172.1 (2)	O1W—Er1—O8—Er2	−86.14 (6)
C16—Er1—C24—O7	−81.57 (14)	O5—Er1—O8—Er2	77.96 (6)
O1W—Er1—C24—O8	−88.92 (11)	O10—Er1—O8—Er2	−0.21 (5)
O5—Er1—C24—O8	63.25 (11)	O4—Er1—O8—Er2	125.61 (6)
O10—Er1—C24—O8	−10.19 (11)	O2—Er1—O8—Er2	−45.07 (9)
O4—Er1—C24—O8	117.73 (11)	O7—Er1—O8—Er2	−173.33 (9)
O2—Er1—C24—O8	−90.86 (14)	O1—Er1—O8—Er2	48.62 (11)
O7—Er1—C24—O8	172.1 (2)	N1—Er1—O8—Er2	−152.25 (6)
O1—Er1—C24—O8	87.9 (2)	C16—Er1—O8—Er2	100.81 (6)
N1—Er1—C24—O8	−166.12 (11)	C8—Er1—O8—Er2	−5.17 (15)
C16—Er1—C24—O8	90.54 (11)	C24—Er1—O8—Er2	−169.00 (14)
C30—C25—C26—C27	−0.7 (3)	O11—C32—O10—Er1	−151.24 (15)
C31—C25—C26—C27	177.9 (2)	C31—C32—O10—Er1	28.7 (3)
C25—C26—C27—C28	−0.4 (4)	Er2—C32—O10—Er1	−139.3 (2)
C26—C27—C28—O12	−179.0 (2)	O11—C32—O10—Er2	−11.97 (19)
C26—C27—C28—C29	1.0 (3)	C31—C32—O10—Er2	167.97 (17)
C27—C28—C29—C30	−0.4 (3)	O1W—Er1—O10—C32	−148.3 (2)
O12—C28—C29—C30	179.6 (2)	O5—Er1—O10—C32	53.5 (2)
C26—C25—C30—C29	1.3 (3)	O4—Er1—O10—C32	47.0 (2)
C31—C25—C30—C29	−177.3 (2)	O2—Er1—O10—C32	−72.9 (2)
C28—C29—C30—C25	−0.7 (4)	O7—Er1—O10—C32	141.0 (2)
C30—C25—C31—C32	75.3 (3)	O1—Er1—O10—C32	−20.8 (2)
C26—C25—C31—C32	−103.3 (3)	N1—Er1—O10—C32	−105.7 (2)
C25—C31—C32—O11	12.2 (3)	O8—Er1—O10—C32	134.9 (2)
C25—C31—C32—O10	−167.75 (19)	C16—Er1—O10—C32	48.1 (2)
C25—C31—C32—Er2	−93.6 (7)	C8—Er1—O10—C32	−47.2 (2)
O8—Er2—C32—O11	−168.55 (12)	C24—Er1—O10—C32	139.9 (2)
O14—Er2—C32—O11	62.15 (12)	O1W—Er1—O10—Er2	76.92 (6)
O2W—Er2—C32—O11	−90.84 (12)	O5—Er1—O10—Er2	−81.30 (6)
O13—Er2—C32—O11	116.21 (12)	O4—Er1—O10—Er2	−87.77 (8)
O16—Er2—C32—O11	−95.36 (14)	O2—Er1—O10—Er2	152.40 (6)
O17—Er2—C32—O11	69.6 (2)	O7—Er1—O10—Er2	6.29 (8)
O10—Er2—C32—O11	168.01 (19)	O1—Er1—O10—Er2	−155.58 (6)
N3—Er2—C32—O11	−12.46 (13)	N1—Er1—O10—Er2	119.59 (11)
C40—Er2—C32—O11	89.12 (12)	O8—Er1—O10—Er2	0.19 (4)
O8—Er2—C32—O10	23.43 (11)	C16—Er1—O10—Er2	−86.64 (7)
O14—Er2—C32—O10	−105.86 (11)	C8—Er1—O10—Er2	178.08 (6)
O2W—Er2—C32—O10	101.15 (11)	C24—Er1—O10—Er2	5.17 (6)
O13—Er2—C32—O10	−51.81 (11)	O8—Er2—O10—C32	−154.36 (12)
O16—Er2—C32—O10	96.63 (13)	O14—Er2—O10—C32	73.09 (11)
O17—Er2—C32—O10	−98.43 (18)	O2W—Er2—O10—C32	−71.40 (11)
O11—Er2—C32—O10	−168.01 (19)	O13—Er2—O10—C32	125.92 (12)
N3—Er2—C32—O10	179.52 (11)	O16—Er2—O10—C32	−118.65 (12)
C40—Er2—C32—O10	−78.89 (11)	O17—Er2—O10—C32	139.16 (13)
O8—Er2—C32—C31	−56.6 (7)	O11—Er2—O10—C32	6.59 (11)
O14—Er2—C32—C31	174.1 (7)	N3—Er2—O10—C32	−0.59 (13)
O2W—Er2—C32—C31	21.1 (7)	C40—Er2—O10—C32	99.09 (11)
O13—Er2—C32—C31	−131.8 (7)	C48—Er2—O10—C32	−158.87 (15)
O16—Er2—C32—C31	16.6 (7)	O8—Er2—O10—Er1	−0.21 (5)
O17—Er2—C32—C31	−178.5 (6)	O14—Er2—O10—Er1	−132.76 (6)
O10—Er2—C32—C31	−80.0 (7)	O2W—Er2—O10—Er1	82.75 (6)
O11—Er2—C32—C31	112.0 (7)	O13—Er2—O10—Er1	−79.93 (6)
N3—Er2—C32—C31	99.5 (7)	O16—Er2—O10—Er1	35.50 (10)
C40—Er2—C32—C31	−158.9 (7)	O17—Er2—O10—Er1	−66.68 (12)
C38—C33—C34—C35	−0.5 (3)	O11—Er2—O10—Er1	160.75 (8)
C39—C33—C34—C35	178.9 (2)	N3—Er2—O10—Er1	153.57 (6)
C33—C34—C35—C36	0.5 (3)	C40—Er2—O10—Er1	−106.75 (6)
C34—C35—C36—O15	179.8 (2)	C48—Er2—O10—Er1	−4.71 (17)
C34—C35—C36—C37	−0.4 (3)	C32—Er2—O10—Er1	154.16 (14)
O15—C36—C37—C38	180.0 (2)	O10—C32—O11—Er2	11.82 (19)
C35—C36—C37—C38	0.2 (3)	C31—C32—O11—Er2	−168.11 (19)
C34—C33—C38—C37	0.3 (3)	O8—Er2—O11—C32	12.71 (13)
C39—C33—C38—C37	−179.07 (19)	O14—Er2—O11—C32	−113.00 (12)
C36—C37—C38—C33	−0.1 (3)	O2W—Er2—O11—C32	80.53 (12)
C38—C33—C39—C40	−114.8 (2)	O13—Er2—O11—C32	−66.79 (12)
C34—C33—C39—C40	65.9 (3)	O16—Er2—O11—C32	118.93 (12)
C33—C39—C40—O14	172.71 (19)	O17—Er2—O11—C32	−149.13 (11)
C33—C39—C40—O13	−8.9 (3)	O10—Er2—O11—C32	−6.71 (11)
O8—Er2—C40—O14	−170.85 (12)	N3—Er2—O11—C32	167.40 (13)
O2W—Er2—C40—O14	−80.58 (19)	C40—Er2—O11—C32	−89.90 (12)
O13—Er2—C40—O14	−178.7 (2)	C48—Er2—O11—C32	162.69 (13)
O16—Er2—C40—O14	102.43 (13)	O14—C40—O13—Er2	1.3 (2)
O17—Er2—C40—O14	92.81 (13)	C39—C40—O13—Er2	−177.12 (18)
O10—Er2—C40—O14	−106.44 (13)	O8—Er2—O13—C40	−172.07 (13)
O11—Er2—C40—O14	−55.10 (13)	O14—Er2—O13—C40	−0.73 (11)
N3—Er2—C40—O14	20.19 (13)	O2W—Er2—O13—C40	−136.17 (13)
C48—Er2—C40—O14	95.32 (13)	O16—Er2—O13—C40	117.35 (12)
C32—Er2—C40—O14	−80.69 (13)	O17—Er2—O13—C40	83.91 (12)
O8—Er2—C40—O13	7.85 (13)	O10—Er2—O13—C40	−102.79 (12)
O14—Er2—C40—O13	178.7 (2)	O11—Er2—O13—C40	−58.54 (12)
O2W—Er2—C40—O13	98.12 (18)	N3—Er2—O13—C40	23.50 (15)
O16—Er2—C40—O13	−78.87 (13)	C48—Er2—O13—C40	98.51 (13)
O17—Er2—C40—O13	−88.49 (13)	C32—Er2—O13—C40	−81.93 (12)
O10—Er2—C40—O13	72.26 (12)	O13—C40—O14—Er2	−1.3 (2)
O11—Er2—C40—O13	123.60 (12)	C39—C40—O14—Er2	177.16 (17)
N3—Er2—C40—O13	−161.11 (12)	O8—Er2—O14—C40	11.55 (15)
C48—Er2—C40—O13	−85.98 (12)	O2W—Er2—O14—C40	142.71 (12)
C32—Er2—C40—O13	98.01 (12)	O13—Er2—O14—C40	0.73 (12)
C46—C41—C42—C43	−0.7 (3)	O16—Er2—O14—C40	−98.32 (13)
C47—C41—C42—C43	177.5 (2)	O17—Er2—O14—C40	−80.18 (13)
C41—C42—C43—C44	−1.0 (4)	O10—Er2—O14—C40	72.32 (13)
C42—C43—C44—O18	−178.9 (2)	O11—Er2—O14—C40	120.90 (13)
C42—C43—C44—C45	1.9 (4)	N3—Er2—O14—C40	−159.64 (14)
O18—C44—C45—C46	179.6 (2)	C48—Er2—O14—C40	−90.23 (13)
C43—C44—C45—C46	−1.2 (4)	C32—Er2—O14—C40	97.35 (13)
C42—C41—C46—C45	1.4 (3)	O17—C48—O16—Er2	7.7 (2)
C47—C41—C46—C45	−176.8 (2)	C47—C48—O16—Er2	−171.02 (16)
C44—C45—C46—C41	−0.5 (4)	O8—Er2—O16—C48	−114.09 (12)
C46—C41—C47—C48	107.5 (3)	O14—Er2—O16—C48	18.12 (14)
C42—C41—C47—C48	−70.6 (3)	O2W—Er2—O16—C48	165.19 (12)
C41—C47—C48—O16	58.9 (3)	O13—Er2—O16—C48	−46.34 (13)
C41—C47—C48—O17	−119.8 (2)	O17—Er2—O16—C48	−4.30 (11)
O8—Er2—C48—O16	64.11 (12)	O10—Er2—O16—C48	−147.09 (11)
O14—Er2—C48—O16	−165.33 (11)	O11—Er2—O16—C48	127.40 (12)
O2W—Er2—C48—O16	−14.59 (12)	N3—Er2—O16—C48	79.14 (12)
O13—Er2—C48—O16	139.39 (11)	C40—Er2—O16—C48	−15.95 (14)
O17—Er2—C48—O16	172.3 (2)	C32—Er2—O16—C48	169.60 (11)
O10—Er2—C48—O16	68.25 (19)	O16—C48—O17—Er2	−7.41 (19)
O11—Er2—C48—O16	−89.06 (16)	C47—C48—O17—Er2	171.30 (17)
N3—Er2—C48—O16	−93.83 (12)	O8—Er2—O17—C48	74.20 (12)
C40—Er2—C48—O16	166.95 (11)	O14—Er2—O17—C48	−157.61 (13)
O8—Er2—C48—O17	−108.23 (12)	O2W—Er2—O17—C48	−8.57 (15)
O14—Er2—C48—O17	22.32 (13)	O13—Er2—O17—C48	146.13 (13)
O2W—Er2—C48—O17	173.07 (12)	O16—Er2—O17—C48	4.25 (11)
O13—Er2—C48—O17	−32.95 (13)	O10—Er2—O17—C48	132.86 (12)
O16—Er2—C48—O17	−172.3 (2)	O11—Er2—O17—C48	−122.34 (13)
O10—Er2—C48—O17	−104.09 (18)	N3—Er2—O17—C48	−79.06 (12)
O11—Er2—C48—O17	98.60 (15)	C40—Er2—O17—C48	174.49 (13)
N3—Er2—C48—O17	93.83 (13)	C32—Er2—O17—C48	−165.18 (15)

### Hydrogen-bond geometry (Å, °)

**Table:**

*D*—H···*A*	*D*—H	H···*A*	*D*···*A*	*D*—H···*A*
O3—H3B···O12^i^	0.82	1.93	2.742 (3)	169
O6—H6B···O3W^ii^	0.82	1.86	2.650 (3)	160
O9—H9A···O17^iii^	0.82	1.86	2.674 (2)	173
O12—H12···O11^iv^	0.82	1.94	2.751 (2)	168
O15—H15C···O6^v^	0.82	1.90	2.716 (3)	174
O18—H18B···O9^ii^	0.82	1.95	2.768 (3)	174
O2W—H2WA···O5	0.83 (4)	1.97 (2)	2.732 (2)	151 (3)
O2W—H2WB···N2^ii^	0.81 (2)	2.06 (2)	2.839 (2)	162 (4)
O3W—H3WB···O3	0.82 (4)	1.99 (4)	2.793 (3)	167 (4)
O1W—H1WA···O13	0.83 (4)	1.96 (2)	2.7333 (19)	156 (3)
O1W—H1WB···N4^i^	0.83 (2)	1.96 (2)	2.781 (2)	171 (3)
O3W—H3WA···O1^vi^	0.84 (4)	1.94 (2)	2.778 (3)	176 (4)

Symmetry codes: (i) *x*, *y*+1, *z*; (ii) *x*, *y*−1, *z*; (iii) −*x*, −*y*+1, −*z*; (iv) −*x*, −*y*, −*z*+1; (v) *x*−1, *y*+1, *z*; (vi) −*x*+1, −*y*+1, −*z*+1.
